# Predicting seizure freedom in the postpartum period: Findings from the Maternal Outcomes and Neurodevelopmental Effects of Antiepileptic Drugs study

**DOI:** 10.1002/epi.70288

**Published:** 2026-05-20

**Authors:** Emma C. Osterhaus, Wesley T. Kerr, Kimford J. Meador, Jacqueline A. French, Angela K. Birnbaum, P. Emanuela Voinescu, Elizabeth Gerard, Page B. Pennell

**Affiliations:** ^1^ Department of Neurology University of Pittsburgh School of Medicine Pittsburgh Pennsylvania USA; ^2^ Department of Psychiatry University of Pittsburgh Pittsburgh Pennsylvania USA; ^3^ Department of Biomedical Informatics University of Pittsburgh Pittsburgh Pennsylvania USA; ^4^ Department of Neurology & Neurological Sciences Stanford University School of Medicine Palo Alto California USA; ^5^ Comprehensive Epilepsy Center New York University Grossman School of Medicine New York New York USA; ^6^ University of Minnesota College of Pharmacy Minneapolis Minnesota USA; ^7^ Brigham and Women's Hospital–Mass General Brigham Harvard Medical School Boston Massachusetts USA; ^8^ Northwestern University Feinberg School of Medicine Chicago Illinois USA

**Keywords:** epilepsy, L‐relationship, partum, pregnancy, seizure, seizure change

## Abstract

**Objective:**

This study was undertaken to evaluate whether seizure freedom in pregnancy predicts seizure freedom in the postpartum period in women with epilepsy (WWE). Prior studies have shown that seizure freedom prior to conception strongly predicts seizure freedom during pregnancy. The postpartum period is considered especially vulnerable to recurrent seizures given rapid hormonal changes, shifting antiseizure medication pharmacokinetics, and disrupted sleep. There is a lack of sufficient data on whether seizure freedom during pregnancy predicts postpartum outcomes.

**Methods:**

Pregnant WWE (aged 14–45 years) were enrolled at <20 weeks gestation in the prospective Maternal Outcomes and Neurodevelopmental Effects of Antiepileptic Drugs study. Participants completed electronic daily seizure and medication diaries with study visits each trimester, and at 6–12 weeks and 6 and 9 months postpartum. Participants also reported retrospective seizure frequency for the 9 months preconception. We used logistic regression to assess whether seizure freedom during pregnancy predicted seizure freedom postpartum, with additional analyses by seizure types. In participants with seizures during pregnancy, we evaluated median percent change in seizure frequency.

**Results:**

This analysis included 331 pregnant WWE. Overall, 60.7% were seizure‐free during pregnancy and 61.0% postpartum. Seizure freedom during pregnancy strongly predicted seizure freedom postpartum (odds ratio [OR] = 6.78, 95% confidence interval [CI] = 4.15–11.1, *p* < .0001), with a predictive value of 78% (95% CI = 72%–84%). Retrospectively reported preconception seizure freedom was also independently associated with postpartum seizure freedom (OR = 5.84, 95% CI = 3.57–9.58, *p* < .0001). Long‐term seizure freedom during pregnancy and preconception was associated with 85% postpartum seizure freedom, whereas long‐term presence of seizures was associated with only 36% postpartum seizure freedom. There were no significant differences by seizure type. Among participants with seizures during pregnancy, median postpartum seizure frequency declined by 77% (interquartile range = seizure‐free to 31% reduced).

**Significance:**

Our data demonstrated that seizure freedom during pregnancy was a robust predictor of seizure freedom during the postpartum period. These data can be used to counsel patients about seizure risk in the postpartum period.

**Key points**

- Seventy‐eight percent of people who were seizure‐free in pregnancy maintained seizure freedom postpartum.
- Seizure freedom both before and during pregnancy was associated with 85% postpartum seizure freedom.
- Among women with seizures in pregnancy, median postpartum seizure frequency declined by 77%.
- Higher seizure frequency in pregnancy was not associated with greater percent reductions in postpartum seizure frequency.
- Variability of seizure frequency during pregnancy was similar to other cohorts.

## 1 INTRODUCTION

More than one quarter of people with epilepsy may become pregnant.^1^, ^2^ The complex relationship between sex steroid hormones and seizures causes unique challenges in people with epilepsy.^3^ These relationships can be bidirectional as hormones can affect some antiseizure medications (ASMs) and seizures, and ASMs and seizures may have an impact on hormones.^3^, ^4^, ^5^

In women with epilepsy (WWE), pregnancy becomes a vulnerable time where extra caution needs to be taken, including more intensive monitoring and possible modification of ASMs.^6^ When planning pregnancy, people with epilepsy can be reassured that seizure freedom for at least 9 months preconception is highly predictive of seizure freedom during pregnancy.^7^, ^8^

The postpartum phase is a particularly vulnerable period due to sleep disruption, stress, changing hormones, and ASM concentrations, often leading to the need for rapid and complex dosing regimen changes.^6^, ^9^, ^10^ Newborn safety is also of great concern.^11^ Due to fear of recurrent seizures, many people with epilepsy are fearful of holding their newborn or parenting without another adult present. No studies have used data from prospective seizure diaries to inform counseling about postpartum seizure precautions based on seizures experienced before delivery.

Our primary goal was to use prospectively collected seizure diaries from a well‐controlled observational study to guide data‐informed counseling about postpartum seizures. By reanalyzing seizure diaries from the Maternal Outcomes and Neurodevelopmental Effects of Antiepileptic Drugs (MONEAD) study, we evaluated whether characteristics of seizure freedom and seizure frequency before delivery were associated with postpartum seizure freedom and seizure frequency. Additionally, for women with who were not seizure‐free, we used a data‐driven approach to estimate the typical variability in seizure frequency to give more information about what a meaningful change in their own seizure frequency would be, thus helping to provide knowledge for the postpartum period.

## 2 MATERIALS AND METHODS

Pregnant WWE, aged 14–45 years, enrolled at ≤20 weeks gestational age (GA) in the longitudinal, prospective MONEAD cohort study were included in this analysis.^7^ Exclusion criteria included women with a GA > 20 weeks at enrollment, exposure to non‐ASM teratogens, the detection of major congenital malformations, or a history of genetic disorder, intelligence quotient < 70, and major medical illness.^7^ Participants with functional neurologic disorder were also excluded. MONEAD also included control groups of nonpregnant WWE and healthy pregnant women, who were not included in this analysis. All participants consented for enrollment in MONEAD. This reanalysis of MONEAD data was approved with a waiver of additional consent by the University of Pittsburgh Institutional Review Board (STUDY24020010).

MONEAD recorded information about the participant's seizure types and epilepsy syndrome, as well as demographic and other clinical data. At the first visit of the study, demographic information was obtained, which included detailed epilepsy histories. Participants were asked about age at onset, seizure type, and prior imaging. The site investigators classified seizures and epilepsy syndromes based on the National Institute of Neurological Disorders and Stroke criteria. Participants' seizures were classified according to the International League Against Epilepsy (ILAE)'s 2010 classification of seizures and epilepsy and categorized as focal, generalized, or unspecified onset (Table S1). A semiology core committee for epilepsy classification consisting of expert epileptologists validated the seizure types in participants. Other than epilepsy and seizure information, MONEAD recorded information about age, self‐identified race, ethnicity, breastfeeding, ASM type(s) and dose, maternal outcomes, and neurodevelopmental outcomes. Treatment regimens, including dosing, were managed by each participant's medical team and were not altered for the study.

**FIGURE 1:**
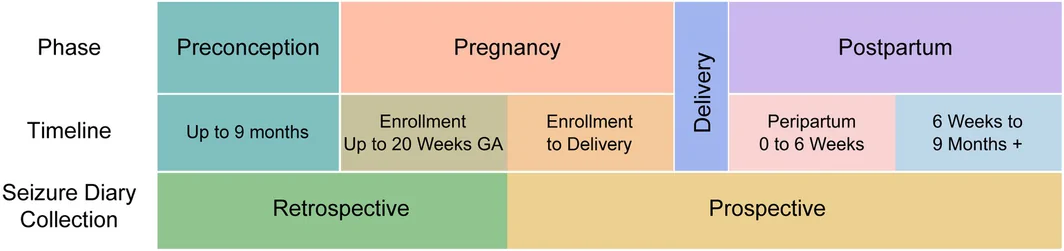
Study design. Study timeline spans preconception, pregnancy, delivery, and postpartum, with pregnancy and postpartum subdivided into clinically relevant observation windows. Data collected during preconception and pregnancy were retrospective, whereas postpartum data were collected prospectively. GA, gestational age.

Seizure frequency was recorded prospectively with an electronic daily seizure and medication diary from the date of enrollment through 9 months postpartum (Figure 1). Study visits occurred approximately every 3 months after enrollment, including a birth visit, to verify diary entries and for other study protocol procedures.^7^ In addition to prospective seizure diaries, women provided retrospective seizure diaries from enrollment back to conception, as well as preconception. Women reported their historical epilepsy type(s), how many seizures occurred from conception to enrollment, and how many seizures occurred in the 9 months prior to pregnancy. The retrospective data during pregnancy (from conception until enrollment) was only used in analyses involving looking at the preconception state.

This analysis focuses on seizure frequency reported during the phases recorded either prospectively or retrospectively. For analysis, we separated prospective phases as (1) postpartum including peripartum (delivery to 9 months), (2) postpartum excluding peripartum (6 weeks postpartum to 9 months), and (3) pregnancy (enrollment [up to 20 weeks GA] through birth). Retrospective phases included (4) early pregnancy (conception through enrollment) and (5) preconception (9 months prior to conception).

### 2.1 Association of pregnancy with postpartum seizure freedom

Due to the clinical importance of seizure freedom, our first analysis focused on seizure freedom and did not evaluate numerical seizure frequency. Seizure freedom was defined as total absence of all seizure types during each prospective or retrospective phase. All analyses included the peripartum period unless its exclusion is explicitly stated.

Using logistic regression, we evaluated the association between postpartum seizure freedom and characteristics of seizures prior to the postpartum period. To build upon prior work, we evaluated associations with seizure freedom during pregnancy based upon prospective diaries, then evaluated the potential association of long‐term seizure freedom by including retrospective pregnancy and preconception diaries. The study used terminology from the 2017 ILAE Classification of Seizure Types. To further characterize these associations, we also evaluated the impact of seizure type and seizures that were focal versus generalized onset. Convulsive seizures included focal to bilateral tonic–clonic (FBTC), generalized tonic–clonic (GTC), and unclassified epilepsy type with convulsions. For this article, we have translated that terminology to the 2025 classification of epileptic seizures.^12^ We defined “convulsive” seizures as focal, generalized, or unclassified seizures with an observable motor component that corresponds to either elementary or complex motor phenomena as defined in the 2025 classification.

As a sensitivity analysis, we evaluated whether these associations differed when postpartum excluded the peripartum phase (birth to 6 weeks postdelivery). We summarize these associations by reporting odds ratios (ORs), Cohen kappa, and predictive statistics of predictive value, sensitivity, and specificity. The 95% confidence intervals (CIs) of predictive statistics were calculated using binomial exact intervals. For all analyses, we considered a *p*‐value less than .05 to be significant. Due to the limited number of potential associated factors, we did not use cross‐validation or other techniques to address potential overfitting.

### 2.2 Associations of recurrent seizures during pregnancy with the median reduction and variability of postpartum seizure frequency

In participants with recurrent seizures during pregnancy (excluding those who were seizure‐free), we also evaluated the median percent reduction in seizure frequency from pregnancy to the postpartum phase. In addition, we evaluated associations between the average and variability (SD) of seizure frequency during both pregnancy and postpartum to investigate what degree of percent reduction in seizure frequency may be statistically meaningful.^13^, ^14^, ^15^

In participants with any type of seizure during their prospective pregnancy seizure diary, we evaluated whether a higher seizure frequency was associated with a different median percent reduction in seizure frequency from pregnancy to postpartum. Consistent with statistical models of seizure frequency change in clinical trials, the percent reduction in seizure frequency was 100% minus postpartum seizure frequency divided by prospective pregnancy seizure frequency.^16^ Due to nonlinearities and potential outliers in percent reduction of seizure frequency, we used rank regression to associate the rank of percent reduction in seizure frequency with the log of seizure frequency in pregnancy.

In addition to median seizure frequency, the variability of seizure frequency allows clinicians to interpret what a statistically meaningful change in seizure frequency may be. Based on previous work from other seizure diary studies (NeuroVista, Seizure Tracker, and Human Epilepsy Project),^13^, ^17^ we evaluated whether there was a linear association between the log of average seizure frequency and the log of the SD of seizure frequency. Using log–log multivariable regression, we evaluated whether that log–log association was different postpartum compared to prospective pregnancy. We also used Wald post hoc statistics to compare the slope of the log–log regression to the value observed in other seizure diary studies, .7, and the Poisson distribution, .5.

## 3 RESULTS

**FIGURE 2:**
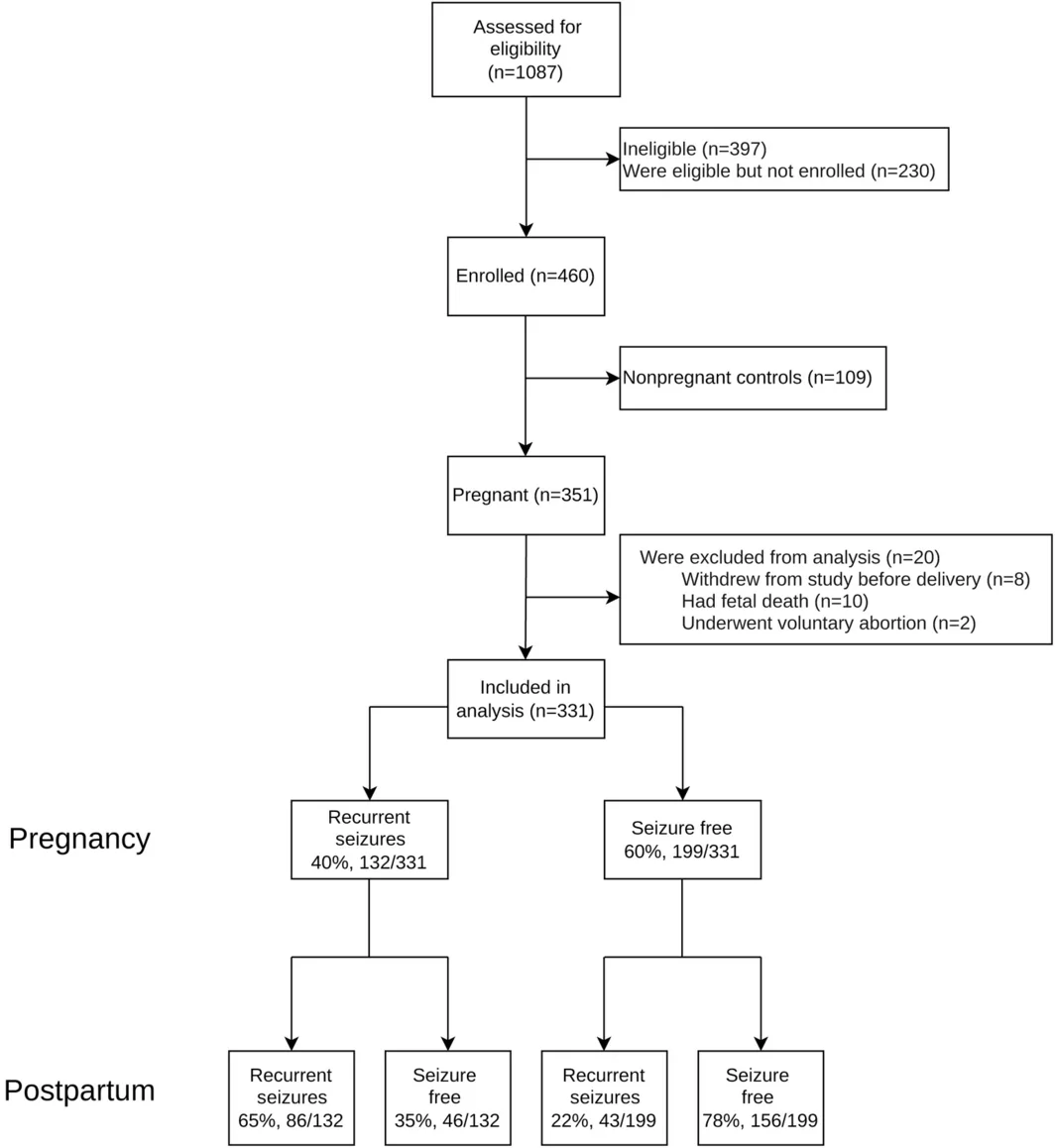
Participant STROBE (Strengthening the Reporting of Observational Studies in Epidemiology) diagram showing screening, enrollment, exclusions, and the final analytic cohort of pregnant participants included in seizure outcome analyses. Pregnancy seizure status (recurrent seizures vs. seizure‐free) and corresponding postpartum outcomes are shown for participants included in the analysis.

MONEAD enrolled 351 pregnant WWE who provided postpartum seizure diary data. Twenty patients were excluded from analysis due to fetal death, voluntary abortion, or withdrawal from the study prior to delivery. This analysis included 331 pregnant WWE (see Figure 2 for STROBE [Strengthening the Reporting of Observational Studies in Epidemiology] flowchart). Table 1 summarizes the characteristics of included participants.

**TABLE 1:** Baseline characteristics, seizure frequency, and ASMs by postpartum seizure outcome.

Characteristic	Seizure‐free postpartum, *n* = 191^a^	Seizures postpartum, *n* = 125^a^
Age, years	31.0 (28.0, 34.0)	31.0 (26.0, 34.0)
Race		
White	164 (86%)	106 (85%)
Black or African American	8 (4.2%)	11 (8.8%)
Asian	7 (3.7%)	4 (3.2%)
Multiracial	8 (4.2%)	2 (1.6%)
Other/unknown	3 (1.6%)	2 (1.6%)
Native Hawaiian or other Pacific Islander	1 (.5%)	0 (0%)
Ethnicity		
Hispanic or Latino	40 (21%)	20 (16%)
Non‐Hispanic or non‐Latino	151 (79%)	105 (84%)
Epilepsy type		
Focal	107 (56%)	77 (62%)
Generalized	63 (33%)	40 (32%)
Unclassified	21 (11%)	8 (6.4%)
Daily seizure frequency, average (95% CI)		
Pregnancy	—	1/2 months (1/9 months, 1/week)
Postpartum	—	1/4 months (1/8 months, 2/month)
ASM regimen^b^		
Monotherapy	161 (84%)	86 (69%)
Polytherapy	30 (16%)	39 (31%)
Choice of ASM		
Levetiracetam	86 (45%)	53 (42%)
Lamotrigine	75 (39%)	46 (37%)
Other	30 (16%)	26 (21%)

Abbreviation: ASM, antiseizure medication.

^a^ Median (Q1, Q3) or *n* (%).

^b^ ASM regimen was associated with postpartum outcome (*p* = .003), with seizures more common in those on polytherapy than monotherapy.

### 3.1 Associations with postpartum seizure freedom

Overall, 60.7% (199/331) participants were seizure‐free during pregnancy and 61.0% (202/331) were seizure‐free postpartum. WWE who were seizure‐free during pregnancy were more likely to be seizure‐free postpartum (OR = 6.78, 95% CI = 4.15–11.1, *p* < .0001). The predictive value of seizure freedom during pregnancy based on postpartum seizure freedom was 78% (95% CI = 72%–84%). Conversely, 35% of participants who had seizures in pregnancy were seizure‐free postpartum (95% CI = 27%–43%). Results excluding the peripartum phase from delivery to 6 weeks were similar and are summarized in Table S2.

**FIGURE 3:**
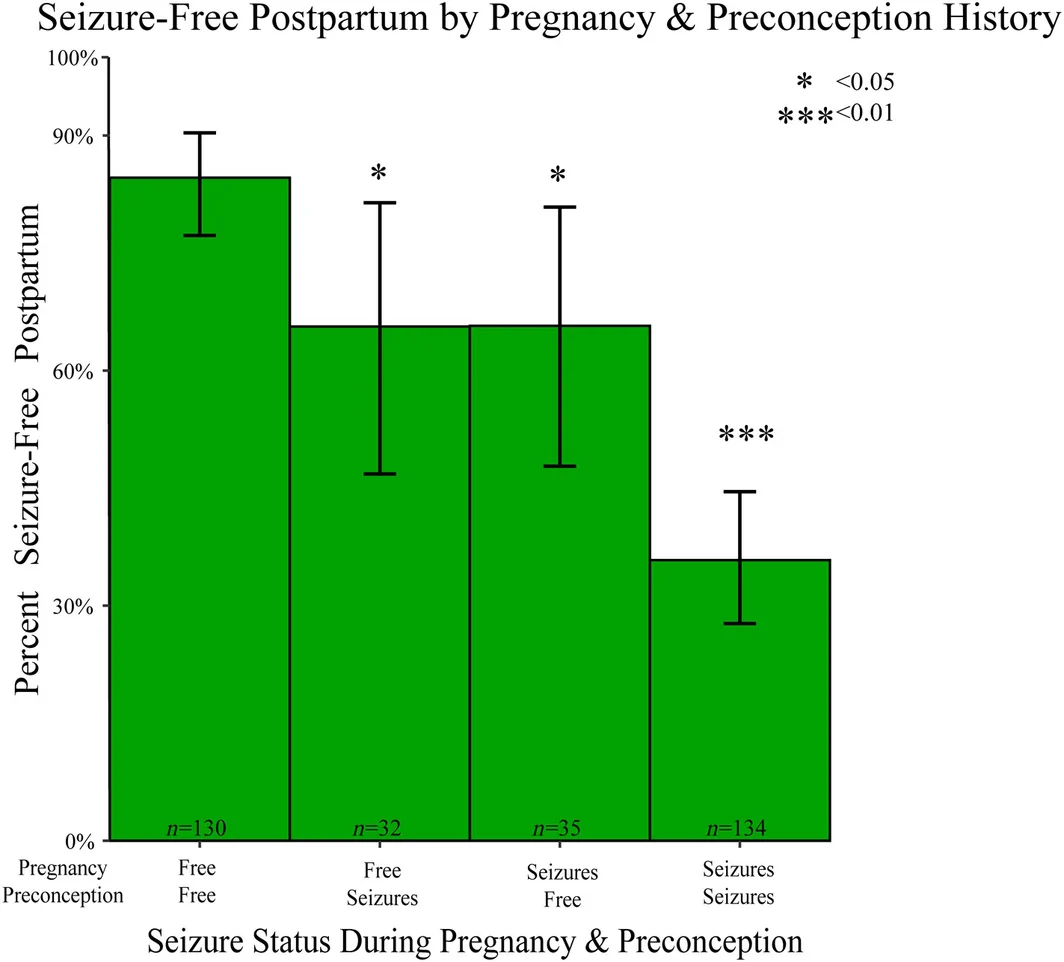
Postpartum seizure freedom rate based on seizure freedom status during pregnancy and preconception. Error bars reflect 95% confidence intervals. *n*, number of participants.

In our evaluation of long‐term seizure freedom, postpartum seizure freedom also was associated with preconception seizure freedom (OR = 5.84, 95% CI = 3.57–9.58, *p* < .0001; Figure 3), conditionally independent of pregnancy seizure freedom (Figure 3). For participants who were seizure‐free during the 9‐month preconception period (irrespective of seizure status in pregnancy), 80.6% were seizure‐free postpartum (95% CI = 75%–87%). In contrast, only 41.6% of participants who had seizures during the 9 months preconception were seizure‐free postpartum (95% CI = 34%–49%; irrespective of seizures in pregnancy).

Long‐term seizure freedom during both 9 months preconception and pregnancy was associated with 85% seizure freedom postpartum (95% CI = 78–91%), whereas seizure occurrence during both pregnancy and preconception had a 36% probability of seizure freedom postpartum (95% CI = 28–44%). Of those who had seizures in pregnancy, 20% were seizure‐free in preconception. The logistic regression analysis revealed that although seizure freedom in both pregnancy and preconception were associated with postpartum seizure freedom, there was no synergistic supra‐additive association of long‐term seizure freedom during both pregnancy and preconception (interaction OR = 1.40, 95% CI = .42–4.70, *p* = .59; Table S3a).

These associations were not significantly different when evaluated for each seizure type (difference between generalized and any seizure type, OR = .97, 95% CI = .43–2.21, *p* = .95). For participants with generalized seizures, 68% were seizure‐free in pregnancy and 61% were seizure‐free postpartum. Among participants with generalized seizures, there was no significant additive association of GTC seizures in pregnancy with any (or convulsive) seizure occurrence postpartum (OR = .8, 95% CI = .2–3.5, *p* = .76), after accounting for the association above with occurrence of any seizure type (OR = 6.8, 95% CI = 4.0–11.8, *p* < .001). For participants with focal seizures, 56% were seizure‐free during pregnancy and 60% were seizure‐free during postpartum. Similarly, in participants with focal seizures, there was no significant additive association between convulsive seizures in pregnancy and any (or convulsive) seizure occurrence postpartum (OR = .9, 95% CI = .3–2.7, *p* = .87), after accounting for the association above with occurrence of any seizure type (OR = 5.1, 95% CI = 2.5–10.6, *p* < .001). We investigated whether convulsions during pregnancy affected seizure freedom postpartum. Convulsions included FBTC seizures, GTC seizures, and convulsive seizures with unclassified onset. Among participants with FBTC seizures, there was a similar association between any or convulsive seizure occurring in pregnancy and any or convulsive seizure occurrence postpartum (any, OR = 5.0, 95% CI = 2.4–10.3, p < .001; change for convulsive, OR = 1.45, 95% CI = .13–33, *p* = .77). Similarly for participants with GTC seizures, there was a similar association between any or GTC seizure occurring in pregnancy and any or GTC seizure occurrence postpartum (any, OR = 6.8, 95% CI = 4.0–11.8; change for GTC, OR = .80, 95% CI = .19–3.4, *p* = .76). When we compared any seizures to seizures with impaired consciousness, we also did not see significant differences.

### 3.2 Seizure characteristics in the subgroup of participants with recurrent seizures during pregnancy

**FIGURE 4:**
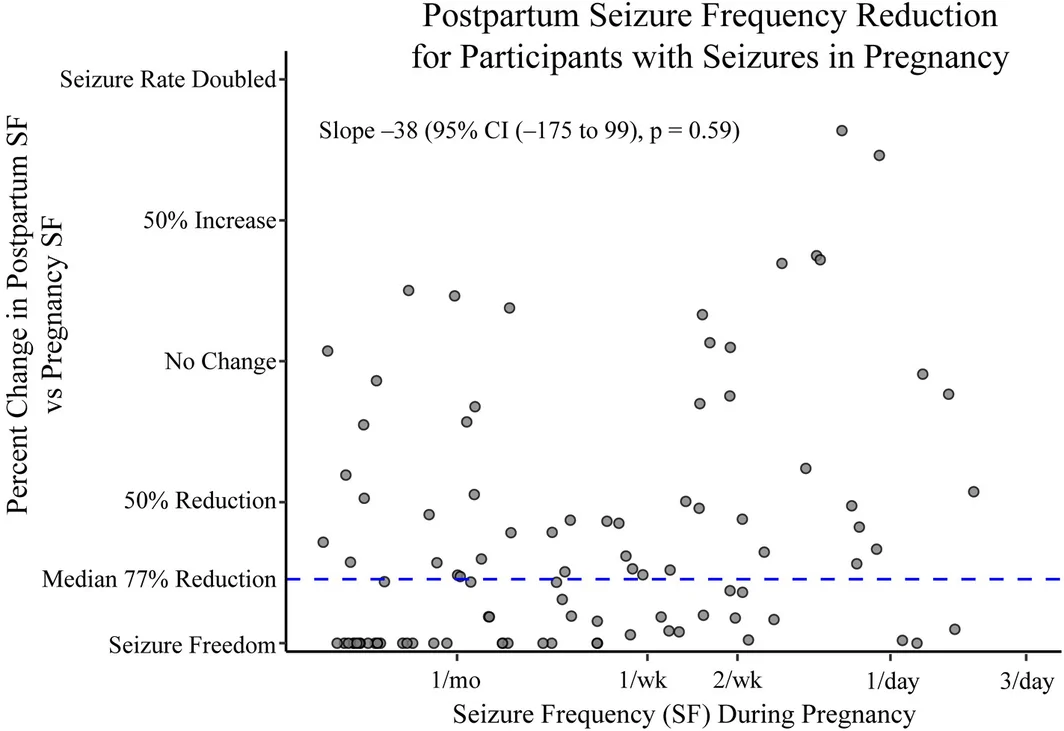
Higher pregnancy seizure frequency was not associated with more or less reduction in postpartum seizure frequency. For participants with seizures during pregnancy, the median percent reduction in postpartum seizure frequency was 77% compared to pregnancy seizure frequency. Each dot reflects one participant. Blue dashed line indicates median. mo, month; SF, seizure frequency; wk, week.

In participants with seizures during pregnancy, the median postpartum reduction in seizure frequency was 77% (interquartile range = −100 to −30.7%; Figure 4). Of the 132 participants with seizures in pregnancy, 18% had an increase in seizure frequency, 12% had a decrease of less than 50%, 35% had postpartum seizures with a 50% or greater decrease, and 35% participants were seizure‐free. Higher seizure frequency in pregnancy was not associated with greater percent reductions in postpartum seizure frequency (*p* = 0.59).

**FIGURE 5:**
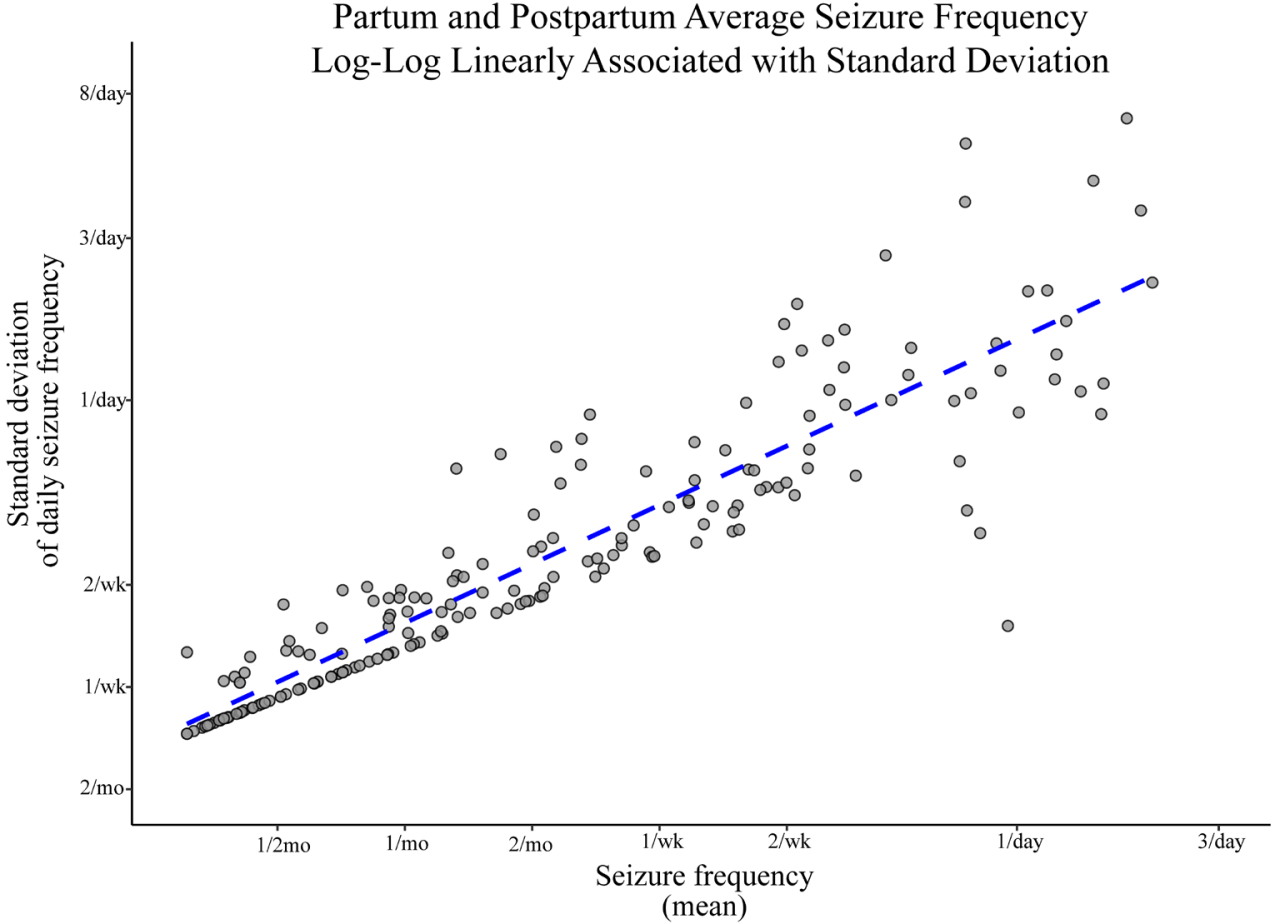
Variability of seizure frequency increased with average seizure frequency. Each dot reflects an individual participant. Dashed blue line indicates the log–log linear association (L‐relationship). mo, month; wk, week.

In our evaluation of the variability of seizure frequency, there was a strong log–log linear association between average and SD of seizure frequency during both pregnancy and the postpartum period (Figure 5). When using daily seizure frequency diaries, the slope of that association was .58 (95% CI = .49–.67; *p* greater than .5 [slope of theoretical Poisson], <.01; *p* less than .7 [slope of nonclinical trial seizure diaries], <.01). When using fortnightly seizure frequency diaries, the slope was .66 (95% CI = .63–.69; *p* greater than .5, <.01; *p* less than .7, <.01). Those associations did not significantly change in the postpartum period compared to the pregnancy period.

For example, when a participant had monthly seizures during pregnancy, the expected 95% CI of their monthly seizure frequency could range from 0 to 2 seizures per month. Alternatively, participants with daily seizures had an expected 95% CI of monthly seizure frequency ranging from weekly seizures to 4.5 seizures per day.

## 4 DISCUSSION

Overall, our data demonstrated that seizure freedom during pregnancy had a robust association with seizure freedom during the postpartum period. Even when participants had seizures during pregnancy, they could have improvements postpartum, because 35% were seizure‐free postpartum and the median reduction in seizure frequency was 77%. When continued seizures were present, our analysis of variability can be used to determine what constituted a statistically meaningful change in seizure frequency. These results can be used to directly counsel patients about seizure precautions and prognosis after delivery based on their seizure control during pregnancy.

These results can reassure WWE who were seizure‐free by informing them that the likelihood of seizures occurring when taking care of their infant is low, although not zero. Our prior publication from the MONEAD cohort demonstrated that 61% of participants were seizure‐free postpartum.^7^ These results show that 78% of participants who were seizure‐free during pregnancy maintained that seizure freedom postpartum. Furthermore, 85% of participants who were seizure‐free long term during both preconception and pregnancy maintained seizure freedom postpartum. In new parents with long‐term recurrent seizures during both pregnancy and preconception, it is appropriate to discuss a postpartum care plan with patients so that both baby and mother can receive the necessary support during this time of change. Because 15% of this population of participants did have seizures, and approximately half of this percentage had convulsive seizures, it remains appropriate to discuss seizure precautions and emphasize medication adherence and other measures to address potential seizures, as discussed on the Epilepsy & Pregnancy Medical Consortium website.^18^

Our study focused on the presence or absence of seizures overall, not the seizure type. Prior studies reported that focal seizures were more likely to worsen during pregnancy compared to generalized onset seizures.^19^, ^20^ Our detailed evaluation of the potential influence of epilepsy type and seizure type demonstrated that changes were similar irrespective of the presence generalized or focal onset seizures, or if their seizures included impaired consciousness or convulsions. Convulsions are of particular importance, as seizures with convulsions are one of the strongest risk factors for sudden unexpected death in epilepsy and other severe consequences of recurrent seizures.^21^, ^22^

To assist in counseling of patients with seizures during pregnancy separately from patients who were seizure‐free, we investigated the change in seizure frequency from pregnancy to postpartum. There was a median reduction of 77% in seizures from pregnancy to postpartum, indicating those with seizures in pregnancy were likely to have seizure reduction postpartum.

These results indicated that seizure frequency did improve over time, which could have been an effect of changing ASM regimens and doses in response to seizures, natural variability in seizure frequency, and increased willingness to change ASM type or dose when the regimen no longer needed to be balanced against risk to the fetus. Additionally, there may be alterations in seizure risk corresponding to hormonal fluctuations occurring between pregnancy, peripartum, and the postpartum period. Therefore, even if patients had seizures in pregnancy, the clinical care provided throughout MONEAD was associated with continual improvement in seizure frequency. These reported seizure frequency change numbers focused on participants who had recurrent seizures in pregnancy and therefore differed from previous reporting of MONEAD, the latter of which also included participants who were seizure‐free during pregnancy.^8^

When a change in seizure frequency occurs during pregnancy and postpartum, our estimates of the variability of seizure frequency can evaluate whether numerical changes reflect a real worsening or improvement in seizure frequency. Especially in these high‐risk periods where ASM modifications can happen quickly in response to a small number of seizures, clinicians need to both accurately and quickly gauge whether seizures have increased, decreased, or stayed the same. Based on similar results, Chiang and colleagues demonstrated that clinicians' accuracy in assessing whether underlying seizure frequency had changed was 75%, including both over‐ and underinterpretation of numerical differences.^23^ By incorporating this understanding of seizure frequency variability, clinical decision support tools improved clinicians' accuracy 88%. In addition to that numerical assessment, clinical decisions to change treatments also consider changes in teratogenic risk of dose changes, seizure type (e.g., tonic–clonic versus focal preserved consciousness), time of day (e.g., nocturnal vs. daytime), and the influence of seizures on quality of life.^24^, ^25^

The slope of the association between the average and SD of seizure frequency was similar to that seen in placebo‐controlled randomized clinical trials and was lower than the other long‐term seizure diary studies of the Human Epilepsy Project, Seizure Tracker, and intracranial monitoring with NeuroVista.^13^ The slope of these associations was higher than the Poisson distribution, which reflected “overdispersion” where there was more variation in seizure count than expected. The similarity of the slope of MONEAD to the clinical trials, both of which had slopes reflecting lower overdispersion than the nonclinical trial diaries, may have been driven by multiple factors.^15^, ^17^ Although ASM dose changes can address recurrent seizures, one goal of ASM management in pregnancy is to maintain ASM concentrations that are comparable to predetermined optimal concentrations within a patient to avoid concentrations known to be inadequate.^8^, ^10^ In contrast, Human Epilepsy Project, Seizure Tracker, and NeuroVista participants may have changed their ASMs and other treatments more frequently or they may have been less diligent about accurately counting seizures, especially for higher seizure frequencies. Although “overdispersion” in seizure count can be caused by unaccounted for modifications of ASM and inaccuracies in seizure counting, it also can be caused by other systematic patterns in seizures such as catamenial and other multiday seizure cycles.

Our approach using MONEAD data to evaluate how seizure information prior to delivery can inform counseling of postpartum seizure control has important limitations. In a sensitivity analysis, we demonstrated that these conclusions did not change when the postpartum period included or excluded the first 6 weeks after delivery, as this is a sensitive time of hormonal change. As is common in studies of patient‐reported diaries, it was challenging to determine nonadherence to diary when patients reported seizure freedom. The participants in MONEAD were followed and managed by local subspecialty experts in epilepsy during pregnancy, which likely included counseling about sleep hygiene, partner support, medication adherence, active monitoring of serum ASM levels, and multiple modifications of ASM doses throughout the study, and therefore, patients may have experienced a lower change in seizure frequency than a more generalized population.^8^, ^10^ Participants in MONEAD were 83% White and 71% college educated; therefore, subsequent studies are needed to understand these patterns in more diverse populations with broader ranges of socioeconomic status and clinical providers. Although this discussion emphasizes how seizures during pregnancy predicted postpartum seizure control based on prospective seizure diaries, preconception seizures also provided valuable information, even though they were temporally separated and based on retrospective seizure diaries.

## 5 CONCLUSIONS

Seizure freedom in pregnancy and preconception was a reliable indicator of postpartum seizure freedom. That association was observed irrespective of the type of epilepsy or type of seizure. In participants who were not seizure‐free during pregnancy, continued subspecialty care was associated with a 35% seizure freedom rate and, if not seizure‐free during pregnancy, 77% median seizure frequency reduction. These results could be used to provide individualized counseling to pregnant patients about what they could expect for seizure frequency postpartum based on how many seizures they had during and before pregnancy.

## AUTHOR CONTRIBUTIONS

Emma C. Osterhaus, Wesley T. Kerr, and Page B. Pennell contributed to hypothesis generation, data analysis, interpretation of results, and drafting of the manuscript. Kimford J. Meador, Jacqueline A. French, Angela K. Birnbaum, P. Emanuela Voinescu, Elizabeth Gerard, and Page B. Pennell contributed to the conception and design of the original study and provided critical revisions and substantive editing of the manuscript. All authors reviewed and approved the final version of the manuscript.

## CONFLICT OF INTEREST STATEMENT

W.T.K. has received compensation as Associate Editor of *Epilepsia*; writes review articles for *Medlink Neurology*; is a paid consultant for SK Life Sciences, UCB Pharmaceuticals, Jazz Pharmaceuticals, Azurity, Acuta, Ventus, Capsida, Epygenix, Biohaven Pharmaceuticals, the Epilepsy Study Consortium, Cerebral Therapeutics, Neurelis, Noema, EpiTel, QurAlis, Neurona, NeuroPace, and Rapport; has collaborative or data use agreements with Eisai, Janssen, Johnson & Johnson, Praxis, Radius Health, and GSK; and has been a site investigator for a trial including UCB Pharmaceuticals and Equilibre Pharmaceuticals. P.B.P. has received royalties as a contributing author for *UpToDate*, research support from the National Institutes of Health (NIH), honoraria for grant reviews from the NIH, and honoraria and travel reimbursements for continuing medical education presentations from academic medical centers and professional societies. K.J.M. has received research support from the NIH, Eisai, and Medtronics; the Epilepsy Study Consortium pays K.J.M.'s university for his research consultant time related to Eisai, UCB Pharma, and Xenon Pharmaceuticals. J.A.F. reports receiving grants from Epilepsy Study Consortium/Epilepsy Foundation funded by UCB; consulting fees from Acadia Pharmaceuticals, Access Industries, Acuta Capital Partners, AFASCI, Agrithera, Alterity Therapeutics Limited, Angelini Pharma, Arvelle Therapeutics, Autifony Therapeutics, Axonis, Baergic Bio, Beacon Biosignals, Biogen, Biohaven Pharmaceuticals, BioMarin Pharmaceutical, Bloom Science, BridgeBio Pharma, Bright Minds Biosciences, Camp4 Therapeutics Corporation, Capsida Biotherapeutics, Cerebral Therapeutics, Cerecin, Cerevel, Ceribell, Clinical Education Alliance, Coda Biotherapeutics, Cognizance Biomarkers, Cowen and Company, Crossject, EcoR1 Capital, Eisai, Eliem Therapeutics, Encoded Therapeutics, Engrail, Epalex, Epihunter, Epiminder, Epitel, Equilibre BioPharmaceuticals, Genentech, Greenwich Biosciences, GRIN Therapeutics, GWPharma, iQure Pharma, IQVIA RDS, Janssen Pharmaceutica, Jazz Pharmaceuticals, Knopp Biosciences, Korro Bio, Leal Therapeutics, Lipocine, LivaNova, Longboard Pharmaceuticals, Lundbeck, Marinus, Modulight.bio, Neumirna Therapeutics, Neurelis, Neurocrine, Neuroelectrics USA Corporation, Neurona Therapeutics, Neuronetics, Neuropace, NeuroPro Therapeutics, Neuroventis, Ono Pharmaceutical Co., Otsuka Pharmaceutical Development, Ovid Therapeutics, Paladin Labs, Pfizer, Praxis, PureTech, Rafa Laboratories, Rapport Therapeutics, Receptor Holdings, Rivervest Venture Partners, Sage Therapeutics, SK Life Sciences, Stoke, Supernus, Takeda, Taysha Gene Therapies, Third Rock Ventures, Ventus Therapeutics, Vida Ventures Management, Xenon, and Zogenix; nonfinancial support/travel fees from Angelini Pharma, Biohaven Pharmaceuticals, Cerebral Therapeutics, Clinical Education Alliance, Cowen and Company, Longboard Pharmaceuticals, Neumirna Therapeutics, Neurelis, Neurocrine, NeuroPace, Praxis, Rapport Therapeutics, SK Life Sciences, Takeda, and Xenon; serving as president for Epilepsy Study Consortium Inc., which provides travel reimbursement and salary support to New York University and is funded by the Andrews Foundation, Eisai, Engage, Lundbeck, Pfizer, SK Life Science, Sunovion, UCB, and Vogelstein Foundation; serving as chief medical/innovation officer for the Epilepsy Foundation, which provides travel reimbursement; receiving grants from GW/FACES/One8Foundation and from National Institute of Neurological Disorders and Stroke outside the submitted work; and serving on the editorial boards of *Lancet Neurology* and *Neurology Today*. E.G. has received support for clinical trials from Stanford University and Eisai as well as Xenon. She has served on advisory boards for Xenon, Stoke, and Jazz Pharmaceuticals. She receives royalties from *UpToDate*. A.K.B. has received research support from the NIH, UCB Pharma, and Vireo Health and is a coinventor on a patent for intravenous carbamazepine. The other authors have no conflicts of interest to disclose. We confirm that we have read the Journal's position on issues involved in ethical publication and affirm that this report is consistent with those guidelines.

## Supporting information


**FIGURE S1.** Postpartum seizure frequency reduction relative to preconception baseline. For participants with seizures during pregnancy, the percent change in seizure frequency from the preconception period to the postpartum period is shown. Higher preconception seizure frequency was not associated with the degree of change in postpartum seizure frequency. The blue dotted line represents the median percent reduction. Each dot reflects one participant. mo, month; wk, week.
**TABLE S1.** Grouping of seizures into convulsive, impaired awareness, and neither based on epilepsy type. The proportion (%) reflects the number of participants with a specific seizure type divided by the total number of participants with any epilepsy type. Participants could have more than one seizure type. *n*, number of patients.
**TABLE S2.** Association of pregnancy seizure freedom with postpartum seizure freedom (excluding peripartum, delivery–6 weeks).
**TABLE S3a.** Logistic regression of pregnancy and preconception seizure freedom predicting postpartum seizure freedom.
**TABLE S3b.** L‐relationship between average and SD of seizure frequency.

## Data Availability

All data included in these analyses will be shared as anonymized data via request from any qualified investigator.
