# Supplementary material for: Predicting seizure freedom in the postpartum period: Findings from the Maternal Outcomes and Neurodevelopmental Effects of Antiepileptic Drugs study
Source: Epilepsia. 2026 May 20;67(8):4109–20. doi: 10.1002/epi.70288 (PMC13525580; doi:10.1002/epi.70288)
Supplement: Supplementary file 1 [file EPI-67-4109-s001.docx]

**Supplemental Table 1**: Grouping of seizures into convulsive, impaired awareness, and neither based on epilepsy type. The proportion, %, reflects the number of participants with a specific seizure type divided by the total number of participants with any epilepsy type. Participants could have more than one seizure type. Abbreviations: number of patients (n).

|  | Epilepsy Types | | | | | | | | |
| --- | --- | --- | --- | --- | --- | --- | --- | --- | --- |
|  | Generalized n=103 | | | Focal | | | Unclassified | | |
|  | Seizure Type | n | % | Seizure Type | n | % | Seizure Type | n | % |
| Convulsive  and Impaired Awareness | Generalized tonic clonic | 99 | 29.9% | Focal bilateral tonic clonic convulsion | 166 | 50.2% | Seizure with convulsions | 24 | 7.3% |
| Impaired Awareness  Only | Typical absence | 37 | 11.2% | With impairment of consciousness | 116 | 35.1% | Seizure type without convulsions, with impaired awareness | 3 | 0.9% |
|  | Atypical absence | 11 | 3.3% |  |  |  |  |  |  |
|  | Myoclonic absence | 4 | 1.2% |  |  |  |  |  |  |
| Neither | Eyelid myoclonia | 1 | 0.3% | With observable motor or autonomic components | 38 | 11.5% | Seizure type without convulsions or impaired awareness | 8 | 2.4% |
|  | Myoclonic tonic | 2 | 0.6% | Involving subjective sensory or psychic phenomena | 94 | 28.5% |  |  |  |
|  | Myoclonus not otherwise specified | 47 | 14.2% |  |  |  |  |  |  |

**Supplemental Table 2. Association of Pregnancy Seizure-Freedom with Postpartum Seizure-Freedom (excluding peripartum, delivery–6 weeks)**

| **Pregnancy**  **status** | **Seizure-free postpartum** | **Seizures postpartum** | **Total** | **% Seizure-free postpartum** | **Odds Ratio (95% CI), p-value** |
| --- | --- | --- | --- | --- | --- |
| Seizure-free pregnancy | 167 | 32 | 199 | 84% (167/199) | 8.29 (5.00–14.04)  p<0.001 |
| Recurrent seizures pregnancy | 51 | 81 | 132 | 39% (51/132) |  |

| **Supplemental Table 3a. Logistic regression of pregnancy and preconception seizure-freedom predicting postpartum seizure-freedom** | | | |
| --- | --- | --- | --- |
| Predictor | Estimate | 95% CI (OR) | p-value |
| Intercept (neither seizure-free) | 0.16 | 0.09–0.27 | <0.001 |
| Pregnancy seizure-free | 3.17 | 1.32–7.62 | 0.010 |
| Preconception seizure-free | 2.84 | 1.17–6.93 | 0.022 |
| Interaction (pregnancy × preconception) | 1.40 | 0.42–4.70 | 0.588 |

To describe the overall model fit, the fixed effects accounted for significant variance (R^2^= 0.27). Accounting for heterogeneity by site also accounted for significant variance (I^2^<0.001)

| **Supplemental Table 3b. L-relationship between average and standard deviation of seizure frequency** | | | |
| --- | --- | --- | --- |
| Predictor | Estimate (β) | 95% CI | p-value |
| **Daily** |  |  |  |
| Intercept | 0.415 | 0.324 – 0.506 | <0.001 |
| Log(mean seizure count) | 0.579 | 0.555 – 0.603 | <0.001 |
| Postpartum (vs During pregnancy) | -0.002 | -0.083 – 0.078 | 0.957 |
| **Fortnightly** | | | |
| Intercept | 0.370 | 0.303 – 0.437 | <0.001 |
| Log(mean seizure frequency) | 0.657 | 0.629 – 0.685 | <0.001 |
| Postpartum (vs Pregnancy) | 0.100 | 0.005 – 0.196 | 0.040 |

Daily: To describe the overall model fit, the fixed effects accounted for significant variance (R^2^= 0.900). Accounting for heterogeneity by site also accounted for significant variance (I^2^<0.001). Fortnightly: To describe the overall model fit, the fixed effects accounted for significant variance (R^2^= 0.89). Accounting for heterogeneity by site also accounted for significant variance (I^2^<0.001).

**Supplemental Figure 1: Postpartum Seizure Frequency Reduction relative to Preconception Baseline.** For participants with seizures during pregnancy, the percent change in seizure frequency from the preconception period to the postpartum period is shown. Higher preconception seizure frequency was not associated with the degree of change in postpartum seizure frequency. The blue dotted line represents the median percent reduction. Each dot reflects one participant. Abbreviations: month (mo), week (wk).
